# Validation of International Caries Detection and Assessment System (ICDAS)-II Short Version and Community Caries Index for Assessing Caries and Treatment Needs in Six- to 15-Year-Old Children in Ghaziabad: A Cross-Sectional Study

**DOI:** 10.7759/cureus.91718

**Published:** 2025-09-06

**Authors:** Kirti Jain, Bhuvan D Gupta, Ritu Gupta, Abhinav Bhargava, Sunil K Chaudhary, Hiba Chaudhary

**Affiliations:** 1 Public Health Dentistry, Institute of Technology and Science (ITS) Centre for Dental Studies and Research, Ghaziabad, IND

**Keywords:** child, dental caries, diagnosis, icdas, oral health, prevalence

## Abstract

Introduction

Dental caries remains a prevalent public health concern, particularly among children in developing countries. The assessment of caries experience and treatment needs is essential for formulating effective intervention strategies.

Objectives

To evaluate and validate the caries experience and treatment needs of children aged 6-15 years in Ghaziabad City using the Community Caries Index of Treatment Needs and International Caries Detection and Assessment System (ICDAS)-II.

Methods

A cross-sectional study was conducted among 1200 school children of Ghaziabad city. Data were collected from government and private schools through multistage cluster sampling. Statistical analysis was performed using SPSS version 25.0 (IBM Inc., Armonk, New York), including sensitivity, specificity, likelihood ratio, predictive values, and receiver operating characteristic (ROC) analysis for diagnostic accuracy.

Results

The short ICDAS-II version demonstrated high diagnostic accuracy for moderate to advanced carious lesions (sensitivity 99.6%, specificity 98.6%, weighted kappa 99.0%), although early-stage, non-cavitated lesions were slightly underreported compared to the full ICDAS-II system.

Conclusion

The ICDAS-II short version provides a practical and effective approach to assessing dental caries in children. Integrating standardized tools like ICDAS-II with the Community Caries Index of Treatment Needs into public health policies can enhance caries prevention and treatment. Strengthening community-based interventions and increasing oral health awareness are key to improving dental health outcomes.

## Introduction

The World Health Organization (WHO) defines health as "a state of complete physical, mental, and social well-being and not merely the absence of disease and infirmity" [1]. Oral health is an integral part of overall health, allowing individuals to eat, speak, and interact socially without discomfort or embarrassment. It plays a crucial role in psychosocial well-being by influencing self-esteem, communication, and productivity.

Oral health evolves across the lifespan, beginning in childhood and continuing through old age. Many oral diseases, including dental caries, are preventable when detected early. In children, oral health significantly affects growth, development, academic performance, and social participation [2].

Dental caries is defined by the WHO as the destruction of the enamel layer of the tooth caused by acids produced by bacterial fermentation of dietary sugars. Despite advancements in prevention and treatment, dental caries remains a major public health concern, particularly in developing countries. In India, high rates of dental caries among children highlight the need for effective strategies to monitor, prevent, and treat this condition [3].

The WHO Global Oral Health Status Report (2022) estimates that oral diseases affect approximately 3.5 billion people worldwide, with a substantial majority residing in middle-income countries [4]. Globally, around 514 million children suffer from dental caries in primary teeth, and 2 billion people are affected by caries in permanent teeth. In India, prevalence studies show variation across regions, with a rate of 36.7% among adolescents (13-19 years) [5] and up to 80.92% among children aged 3-15 in Maharashtra [6]. A study conducted in Ghaziabad in 2020 reported a prevalence of 54.6% [7].

Various indices have been developed to assess dental caries, including the Decayed, Missing, and Filled Teeth (DMFT) index, Significant Caries Index (SIC), Nyvad Criteria, Caries Assessment Spectrum and Treatment (CAST), and the International Caries Detection and Assessment System (ICDAS). Among these, the DMFT index remains the most widely used but has notable limitations, such as equal weighting of decayed, missing, and filled teeth and its inability to detect early enamel lesions [8,9].

The ICDAS-II system, introduced in 2005 [10], allows for detailed multi-stage detection of caries, including early lesions. However, its complexity and time-consuming nature limit its widespread use in community health settings. To address this, a short version of ICDAS-II was developed to simplify the process while maintaining diagnostic accuracy [11].

The ICDAS-II short version focuses on fewer tooth surfaces, increasing efficiency in field studies while retaining sensitivity for early caries detection. It offers improved inter- and intra-examiner reliability and is suitable for both clinical research and public health applications. [12]

India faces a significant burden of pediatric dental caries, necessitating reliable and feasible assessment tools for effective public health planning. Ghaziabad, a rapidly urbanizing city in the National Capital Region, offers a diverse socio-economic population ideal for localized research. This study adds to the existing literature by being one of the few to validate the short version of ICDAS-II in a large, urban-rural population of North India. While previous studies have primarily reported caries prevalence or focused on the full ICDAS-II system, our research demonstrates that the simplified version retains excellent diagnostic accuracy and feasibility for large-scale epidemiological surveys. These findings provide critical evidence supporting the integration of simplified caries detection tools into community-based oral health programs and policy planning, especially in resource-constrained settings.

This study aims to validate the ICDAS-II short version for detecting caries and assessing treatment needs in children aged 6-15 years in Ghaziabad, using the Community Caries Index of Treatment Needs and the original ICDAS-II as the gold standard.

## Materials and methods

Study design

A cross-sectional observational study was conducted among schoolchildren aged 6 to 15 years in Ghaziabad, Uttar Pradesh, India, during the 2022-2024 academic years.

Ethical approval and consent

The study protocol was approved by the Institutional Ethics Committee and Review Board. Informed consent was obtained from parents or guardians, and assent was taken from children. Permissions were secured from school authorities prior to the study.

Inclusion and exclusion criteria

Inclusion criteria included children between the ages of 6 and 15 years who were enrolled in the selected schools of Ghaziabad, provided they had obtained written parental consent and gave their own assent to participate. Only healthy children without any systemic conditions that could potentially influence their oral health were considered. We excluded any child who was currently undergoing orthodontic treatment, had congenital oral abnormalities affecting their dentition, or demonstrated uncooperative behavior that would prevent a reliable oral examination. Children with prior dental restorations were included, but restored tooth surfaces were recorded using ICDAS codes for restorations and secondary caries to avoid bias in diagnostic scoring.

Pilot study and sample size estimation

A pilot study was conducted on a small group of schoolchildren to assess feasibility and refine procedures. Based on the pilot, the prevalence of caries was estimated at 54.6%. The sample size was calculated using formulas based on estimated sensitivity (80%), specificity (80%), prevalence (30%), and a 95% confidence level (Z=1.96). The estimated sample size was 1171, rounded to 1200 for the main study.

Sampling procedure

A multistage random sampling method was used. Ghaziabad was divided into five zones: City Zone, Kavi Nagar Zone, Vijay Nagar Zone, Mohan Nagar Zone, and Vasundhara Zone. From each zone, two public and two private schools were randomly selected. From each selected school, 60 students were chosen using a computer-generated random number table from the class-wise student list, with equal representation from the 6-11 and 12-15-year age groups.

Methods of data collection

Data were collected using a pre-designed pro forma with four sections: 1) Demographic details (age, gender, location); 2) Socioeconomic status (Modified Kuppuswamy Scale) [13]; 3) Oral hygiene practices; and 4) Clinical examination using ICDAS-II (full version) 10 and Community Caries Index of Treatment Needs (ICDAS-II short version) [12].

A trained and calibrated dentist performed all examinations using a mouth mirror and a ball-ended explorer. Calibration exercises were conducted prior to data collection on 50 schoolchildren not included in the main study. Inter- and intra-examiner reliability for ICDAS scoring was assessed using weighted kappa statistics, yielding kappa values of 0.91 for inter-examiner and 0.94 for intra-examiner reliability, indicating excellent agreement.

Statistical analysis

Data were analyzed using SPSS v25.0 (IBM Inc., Armonk, New York). Descriptive statistics (mean, percentages, standard deviations) were computed. Based on ICDAS caries scores, children were grouped into three categories: no caries (ICDAS 0), preventive care (scores 1-3), and operative care (scores >3). Agreement between full and short ICDAS versions was evaluated using the weighted Kappa statistic. Sensitivity, specificity, positive and negative predictive values, likelihood ratios, and ROC curves were calculated to assess the diagnostic performance of the short ICDAS version against the full version.

## Results

Descriptive characteristics of the study population, which included a total of 1200 children aged 6-15 years, were evenly distributed between rural 600 (50.0%) and urban 600 (50.0%) settings. The sample consisted of children from different socioeconomic backgrounds, with 368 (30.7%) from the lower-middle and 352 (29.3%) from the upper-middle socioeconomic classes. A slight male predominance was observed, accounting for 616 (51.3%) of the participants. Most children 509 (42.4%) had never visited a dentist, and pain and consultation were the most common reasons for dental visits. Although 890 (74.2%) of children reported cleaning their teeth, only 206 (23.2%) brushed twice daily, and 187 (26.9%) changed their toothbrushes infrequently (less than once every six months). The majority, 262 (37.7%), used a horizontal brushing technique, with other techniques less common, suggesting the need for improved oral hygiene education in this population (Table 1).

**Table 1:** Descriptive results of study population

Variables		N (%)
Age (in years)	6-11 years	600 (50.0)
	12-15 years	600 (50.0)
Gender	Male	616 (51.3)
	Female	584 (48.7)
Location of Residence	Rural	600 (50.0)
	Urban	600 (50.0)
Education	Government	200 (50.0)
	Private	200 (50.0)
Socioeconomic status	Upper (I)	81 (6.8)
	Upper middle (II)	352 (29.3)
	Lower middle (III)	368 (30.7)
	Upper lower (IV)	346 (28.8)
	Lower (V)	58 (4.4)
Dental History		
Last visit to the dentist	Less than 6 months	154 (12.8)
	6–12 months	211 (17.6)
	More than 1 year but less than 2 years	104 (8.7)
	2 years or more but less than 5 years	124 (10.3)
	5 years or more	98 (8.2)
	Never received dental care	509 (42.4)
Reason for your last visit to the dentist	Consultation/advise	307 (44.4)
	Pain or trouble with teeth, gums or mouth	210 (30.4)
	Treatment/ follow-up treatment	90 (13.1)
	Routine Check-up/treatment	75 (10.8)
	Don’t know/don’t remember	9 (1.3)
Oral hygiene practice		
Do you clean your teeth?	Yes	890 (74.2)
	No	310 (25.8)
Do you use toothpaste to clean your teeth?	Yes	720 (80.9)
	No	170 (19.1)
Type of cleaning agent	Fluoridated toothpaste	698(96.9)
	Non-Fluoridated toothpaste	22 (3.1)
Items used to clean their teeth	Toothbrush	696 (78.2)
	Others (Dental floss, Charcoal, Wooden toothpicks, Chewstick/miswak, Plastic toothpicks	194 (21.8)
			Frequency of cleaning teeth	Once a day	573 (64.3)
				2–3 times a month	24 (2.7)
				2–6 times a week	79 (8.9)
Once a month	08 (0.9)		
Twice or more a day	206 (23.2)		
Time taken to brush teeth	<2min	252 (36.2)
	>5min	52 (7.5)
	2 to 5 min	392 (56.3)
Frequency of changing toothbrush	Up to 3 months	157 (22.6)
	3-6 months	352 (50.5)
	more than 6 months	187 (26.9)
Technique for tooth brushing	Horizontal technique	262 (37.7)
	Roll technique	19 (2.7)
	Vertical technique	156 (22.4)
	Combination	259 (37.2)

This study compares the mean ICDAS scores for caries severity using both the full and short versions of the ICDAS system. Both versions showed a consistent decrease in mean scores with increased severity of lesions. However, the short version demonstrated lower sensitivity for early lesions (i.e., ICDAS scores 1-2), which were slightly underreported. This limitation has important implications for preventive care strategies. Under-detection of early non-cavitated lesions may lead to missed opportunities for interventions such as fluoride application, dietary counseling, and oral hygiene education - measures that can arrest or reverse the caries process at its initial stages. Consequently, some children might progress to cavitated lesions requiring operative treatment, increasing the long-term burden on public health systems. Therefore, while the short ICDAS version is efficient for large-scale assessments, it should ideally be supplemented with preventive programs and periodic professional examinations to ensure early lesion detection and timely care (Table 2).

**Table 2:** Comparison of mean ICDAS scores of the children using full and short versions ICDAS - International Caries Detection and Assessment System

	ICDAS full version	ICDAS short version
	Mean ± standard deviation	Mean ± standard deviation
Sound tooth	23.90±2.11	10.5±1.90
First visual change	1.32±0.74	0.31±0.69
Distinct visual change	0.59±0.37	0.05±0.37
Enamel break	0.41±0.11	0.03±0.11
Dentin dark shadow	0.58±0.08	0.01±0.88
Visible dentin	0.95±1.25	0.88±1.18
Extensive cavity	0.25±0.57	0.17±0.57

The sensitivity ranged from 95.9% to 99.6%, indicating that the short version is highly effective at detecting caries. The specificity was also high, ranging from 98.6% to 100%, confirming that the tool can accurately identify healthy teeth. The positive predictive value (PPV) ranged from 93.9% to 100%, and the negative predictive value (NPV) ranged from 98.8% to 99.6%, further reinforcing the reliability of the tool. Receiver operating characteristic (ROC) values approached 99.5%, and the weighted kappa values ranged from 93.8% to 99.9%, indicating excellent agreement with the full ICDAS system, particularly for identifying advanced caries lesions (Table 3).

**Table 3:** Operating characteristics of the short ICDAS to classify treatment needs LR - likelihood ratio; PPV - positive predictive value; NPV - negative predictive value; ROC - receiver operating characteristic;  CI - confidence interval; ICDAS - International Caries Detection and Assessment System

Variables	Non-operative care (ICDAS 0)	Preventive care (ICDAS 1-3)	Operative care (ICDAS >3)
Sensitivity (95%CI)	98.9 (97.7-99.6)	95.9 (92.9-97.9)	99.6 (98.6-99.9)
Specificity (95%CI)	100 (99.4-100)	100 (99.6-100)	98.6 (94.6-98.7)
+LR (95%CI)	-	-	-
-LR (95%CI)	0.01 (0-0.02)	0.04(0.02-0.07)	0 (0-0.02)
Disease Prevalence (95%CI)	47.1 (44.3-50.0)	22.7 (20.4-25.2)	45.7 (42.9-48.6)
PPV (95%CI)	100 (99.3-100)	100 (98.6-100)	93.9 (91.8-95.5)
NPV (95%CI)	99 (97.9-99.5)	98.8 (97.9-99.3)	99.6 (98.7-99.9)
ROC (95%CI)	99.5 (98.9-99.8)	99.08 (98.3-99.5)	96.9 (95.7-97.8)
Weighted Kappa score	99.9%	97.4%	93.8%
P-value	0.000	0.000	0.000

## Discussion

This study aimed to validate the short version of the ICDAS system for caries detection and classification of treatment needs in a sample of 1200 children aged 6-15 years in Ghaziabad, India.

In the present study, an equal number of children from both urban and rural areas were taken, which was comparable to the study conducted by Chandregowda [14] in 2020, where the schools were classified as urban or rural based on the fact that oral health care in rural areas is limited due to a lack of perceived need for dental care.

In the present study, the most common reason for dental visits was consultation (44.4%), followed by a visit due to pain or oral discomfort (30.4%). The relatively low proportion (10.8%) of children visiting the dentist for routine checkups suggests the need for improved awareness and policies promoting preventive oral healthcare. These study results were in accordance with the study conducted by Rambabu [15] in urban India to find out the reasons for use and nonuse of dental services among people visiting a private dental hospital in urban India, where 38% children visited for pain, followed by cavity at 27%.

The present study results found that 74.2% study participant cleaned their teeth, which is important for establishing lifelong oral health habits, whereas 25.8% did not clean their teeth, leading to a higher risk for dental caries, gum disease, and other oral health problems. This was comparable to the study conducted by Mehta et al. [16], where it was found that the majority of the children (71.4%) used a toothbrush and toothpaste for cleaning their teeth. Children who do not use toothpaste may face barriers such as a lack of access to dental products, limited parental awareness, or cultural practices that favor alternative cleaning methods.

The study result represents the distribution of ICDAS II codes among the study population, providing insights into the extent and severity of dental caries. More advanced carious stages, including enamel break (0.41 ± 0.11), dentin dark shadow (0.58 ± 0.08), visible dentin (0.95 ± 1.25), and extensive cavity (0.25 ± 0.57), highlight the progressive nature of untreated dental decay in the study population. The relatively high mean value of sound teeth suggests that a significant proportion of children have healthy dentition, but the presence of advanced lesions (visible dentin and extensive cavity) indicates the need for improved preventive care and timely treatment. These findings underscore the importance of early caries detection through regular dental screenings and the use of tools like ICDAS to classify and monitor caries progression [17].

According to the ICDAS shorter version, a significant number of teeth remain sound; however, there is a notable presence of early-stage carious lesions. The presence of early enamel lesions, with a mean of 0.31 ± 0.69 teeth exhibiting the first visual change in enamel (ICDAS code 1), indicates the initial onset of demineralization. This early stage is critical, as timely intervention can halt or even reverse the progression of dental caries. These study results were comparable with a study conducted by Arangannal [18], which reported a caries prevalence of 47% among schoolchildren aged 11-12 years, with a significant proportion of lesions in the advanced stages (ICDAS codes 5-6).

The present study results provide a comprehensive overview of the diagnostic accuracy of the shorter version of ICDAS in assessing dental caries among school children of Ghaziabad city, among 6- to 15-year-olds. The caries treatment needs were classified into non-operative care, preventive care, and operative care, emphasizing the need for prevention in caries management. In this, the non-operative care aims to diagnose and manage non-cavitated carious lesions as soon as possible to avoid additional tooth damage. Preventive care aimed to limit the exposure of caries risk factors by modifying unhealthy behaviour and building caries resistance by promoting caries prevention programs. Lastly, the operative care seeks to reduce the burden of caries lesions by avoiding pulpal involvement, tooth loss, and simultaneously restoring aesthetics and functioning. Based on this, it can be stated that the simplified version demonstrated good agreement in categorizing children into non-operative care, which is required for managing non-cavitated lesions, whether they are active or not. On the other hand, cavitate lesions require appropriate treatment by the dentist, falling under the operative care of treatment needs. So, this could be used at the community level to manage a larger portion of caries management and make it more cost-effective.

In the shorter version, 10 surfaces of the primary teeth and 12 surfaces from the permanent dentition were taken because they are more prevalent for caries in children and have shown excellent diagnostic accuracy for the treatment needs of the children of Ghaziabad city.

In the present study, to measure the diagnostic accuracy of the shorter version of ICDAS, sensitivity, specificity, likelihood ratio, ROC, and predictive values were used.

The results showed that the ICDAS short version had high sensitivity (95.9%-99.6%) and specificity (98.6%-100%), indicating that it performs exceptionally well in identifying both diseased and sound teeth when compared to the full ICDAS system. These findings are consistent with previous literature that supports the reliability of simplified caries detection tools in large-scale epidemiological surveys [11].

The positive predictive values (93.9%-100%) and negative predictive values (98.8%-99.6%) further emphasize the diagnostic accuracy of the short version, suggesting that it can be a dependable alternative in field settings where time and resources are limited. The ROC values and kappa scores were also notably high, indicating strong agreement and discriminatory power between the two systems. Our study results were in accordance with the study conducted by ElSalhy et al. [12], where the ROC was found to be 90% in primary dentition and 89% in permanent dentition. When comparing the non-operative and operative care, the lowest sensitivity measure was 81.9%. A 100% specificity rate is achieved by the short ICDAS in all comparisons of treatment needs. Furthermore, the NPV was 88% when it came to distinguishing between non-operative and operative categories, while the PPV was 100% when it came to distinguishing between various treatment demands.

Our findings are consistent with international studies validating the ICDAS system. For example, Braga et al. (Brazil, 2009) reported high reliability of ICDAS-II in epidemiological surveys, with weighted kappa values above 0.80, similar to our results [18]. Likewise, ElSalhy et al. (Canada, 2022) proposed a community caries index derived from ICDAS, demonstrating excellent diagnostic accuracy with ROC values exceeding 90%, aligning with the sensitivity and specificity observed in our study [17]. Comparable findings were reported in Europe by Honkala et al. (Finland, 2011) and in the US by Ismail et al. (2007), where ICDAS showed strong diagnostic performance and feasibility for large-scale screenings [13,10]. By validating the short version in a large Indian cohort, our study adds to this global body of evidence, supporting its use in resource-limited settings and large epidemiological surveys worldwide.

Recent systematic reviews have further validated the efficacy of the ICDAS system. For instance, Kapor et al. (2021) assessed the diagnostic performance of various methods for occlusal caries diagnostics, including visual examination, bitewing radiography, and laser fluorescence, in relation to their ability to detect dentin caries under clinical and laboratory conditions [19]. Similarly, Janjic Rankovic et al. (2021) evaluated the diagnostic accuracy and reliability of commonly used caries detection methods for proximal caries diagnostics [20]. Additionally, Ibrahim et al. (2021) compared the diagnostic accuracy of digital radiography and other novel diagnostic tools with visual ICDAS criteria [21].

One of the key strengths of this study is its large sample size and balanced demographic representation across age, gender, socioeconomic status, and residence, which supports the applicability of the findings within Ghaziabad. However, differences in lifestyle, dietary patterns, and healthcare access across other regions of India and globally may limit the generalizability of these results beyond the study population. Additionally, the similarity in mean caries scores between the two systems for advanced lesions suggests that the short version is particularly effective at identifying cases requiring operative treatment, an important clinical consideration.

However, it was observed that the short version slightly underreported early non-cavitated lesions compared to the full ICDAS system. This limitation may be attributed to the simplified criteria used in the short version, which may not capture subtle enamel changes. Other limitations, such as sampling bias and examiner subjectivity, should be acknowledged. Despite these limitations, in contexts where early lesion detection is not the primary focus, such as community screenings or national oral health surveys, the short version still proves to be a valid and efficient tool.

In conclusion, the study strongly supports the use of the ICDAS short version as a reliable, time-saving alternative to the full ICDAS, particularly for large-scale dental health assessments. Further research may explore its application in different age groups and in varied clinical environments to reinforce these findings.

## Conclusions

The short version of ICDAS is an effective tool for detecting caries and classifying treatment needs in children aged 6-15 years. Despite its limitations in underreporting early, non-cavitated lesions, it demonstrates high sensitivity and specificity for identifying teeth requiring preventive or operative treatment. The short ICDAS version is particularly valuable in large-scale dental screenings and community-based public health programs, where simplicity and efficiency are paramount. It should be noted that, while highly practical, the short version is not fully comprehensive, which is acceptable in contexts where rapid assessment is the priority.
